# Feasibility of common, enjoyable game play for assessing daily cognitive functioning in older adults

**DOI:** 10.3389/fneur.2023.1258216

**Published:** 2023-10-12

**Authors:** Nadine Schwab, Chao-Yi Wu, Jake Galler, Thomas DeRamus, Abaigeal Ford, Jessica Gerber, Robert Kitchen, Barnaly Rashid, Misha Riley, Lauren Sather, Xifeng Wang, Cathrine Young, Liuqing Yang, Hiroko H. Dodge, Steven E. Arnold

**Affiliations:** ^1^Neurology, Massachusetts General Hospital, Harvard Medical School, Charlestown, MA, United States; ^2^AbbVie, Inc., North Chicago, IL, United States

**Keywords:** game-based assessments, cognitive monitoring, Alzheimer’s disease and related dementias, endpoints assessment, intensive monitoring, feasibility and acceptability, user experience, trial assessment

## Abstract

**Background:**

Frequent digital monitoring of cognition is a promising approach for assessing endpoints in prevention and treatment trials of Alzheimer’s disease and related dementias (ADRD). This study evaluated the feasibility of the MIND GamePack^©^ for recurrent semi-passive assessment of cognition across a longitudinal interval.

**Methods:**

The MIND GamePack consists of four iPad-based games selected to be both familiar and enjoyable: Word Scramble, Block Drop, FreeCell, and Memory Match. Participants were asked to play 20 min/day for 5 days (100 min) for 4 months. Feasibility of use by older adults was assessed by measuring gameplay time and game performance. We also evaluated compliance through semi-structured surveys. A linear generalized estimating equation (GEE) model was used to analyze changes in gameplay time, and a regression tree model was employed to estimate the days it took for game performance to plateau. Subjective and environmental factors associated with gameplay time and performance were examined, including daily self-reported questions of memory and thinking ability, mood, sleep, energy, current location, and distractions prior to gameplay.

**Results:**

Twenty-six cognitively-unimpaired older adults participated (mean age ± SD = 71.9 ± 8.6; 73% female). Gameplay time remained stable throughout the 4-months, with an average compliance rate of 91% ± 11% (1946 days of data across all participants) and weekly average playtime of 210 ± 132 min per participant. We observed an initial learning curve of improving game performance which on average, plateaued after 22–39 days, depending on the game. Higher levels of self-reported memory and thinking ability were associated with more gameplay time and sessions.

**Conclusion:**

MIND GamePack is a feasible and well-designed semi-passive cognitive assessment platform which may provide complementary data to traditional neuropsychological testing in research on aging and dementia.

## Introduction

Clinical treatment trials and longitudinal observational research studies of cognition in aging, Alzheimer’s disease and related dementias (ADRD) typically rely on infrequent (i.e., annual or semi-annual) cognitive measures as endpoints (1, 2). However, this approach has proven to be inefficient in detecting subtle cognitive changes amidst daily variability (e.g., “good days and bad days”) in performance of older adults with or without cognitive decline (3, 4). With such variability, ADRD trials may require thousands of participants and long durations of follow-up to accurately and reliably determine if significant changes in cognition are present (5). This contributes to the protracted time and enormous expense of bringing a new drug to market. It can take more than 13 years (4, 6) and the field has spent over $42.5 billion since 1995 (7), with few meaningful successes to show (8, 9).

Frequent, or even continuous, passive monitoring through the use of mobile applications, web-based testing, and home-based digital sensors has emerged as a promising alternative for measuring cognition in aging and ADRD trials (10–12). Studies have shown that using such tools in a hypothetical pre-symptomatic AD trial can reduce the required sample size for detecting treatment effects by at least 50% (13, 14). This reduction in sample size is attributed to more frequent and sensitive assessments to detect variability (15), which provides more robust data than infrequent or regular clinical visits. Further, frequently monitoring changes in cognition-related functions helps shorten trial periods by detecting treatment effects early (16). Models suggest continuous monitoring could reduce the cost of developing a new ADRD drug from $5.7 billion to $2 billion (6). Although mobile neuropsychological testing (17) and wearable technologies for activity and sleep (18) have been proposed as possible frequent/continuous monitoring solutions, each of these methods has limitations. Web or mobile neuropsychological tests are derived to mimic more conventional neuropsychological tests but are vulnerable to practice effects and require commitment and concentrated effort by participants, even if designed for ease of use (19, 20). Wrist, ring, or pocket wearables do not measure cognition directly (21–23). These drawbacks create a gap between continuous, passive but obscure proxies of cognitive function and more formalized, active, and directed measurements of neuropsychological functions.

As a compromise to achieve more dense monitoring of cognition without the limitations of repeated neuropsychological testing, our team has developed a game-based solution for semi-passive, daily monitoring of cognitive functioning. The MIND GamePack^©^ is a cognitive function monitoring platform with a front end of familiar and enjoyable games and interfaces and a back end of cloud-based servers and basic analysis tools, making it a complete solution that can be deployed in clinical trials or longitudinal research. Our selected games, including Memory Match (inspired by Concentration/ Memory®), FreeCell (Solitaire) (24–26), Word Scramble (Boggle™), and Block Drop (Tetris®) (27), are popular among older Americans (28, 29). Many Baby Boomers and Generation X’ers are very comfortable with the iPad touch-screen medium, and many engage with digital games for leisure (28, 29). These generations are now reaching an age where they face a higher risk of cognitive decline and dementia. However, their familiarity with consumer electronics, including electronic games, presents an opportunity to implement a game-based solution that appeals to older adults. Such an approach has the potential to generate greater interest and compliance in research and trials (28). Moreover, deploying games to participants’ mobile devices is scalable and inexpensive, and game outcomes have been demonstrated to reflect aspects of players’ cognitive functions, providing direct measurement of cognition (30–32). Games with multiple difficulty levels and different puzzles/configurations within a level may also help reduce the practice effects, fatigue and boredom, and potential administration and data errors observed in formalized cognitive tests (33).

After refining MIND GamePack for appearance, usability, and back-end operations in beta phase testing with older adults, we instituted a Phase I study to evaluate feasibility in a longitudinal setting. Within this paradigm, it was hypothesized a learning curve would plateau for each game for each participant. Our group also wanted to explore how subjective and environmental factors may account for differences in play between daily sessions. The current study assessed the feasibility of the MIND GamePack with a group of cognitively unimpaired older adults who played in their home environments over a four-month period. The study aimed to gain insights into the real-world application of game play as a sensitive and reliable outcome measure for future research in aging, ADRD and other disorders which may impact cognition.

## Materials and methods

### Study design

**Figure 1:**
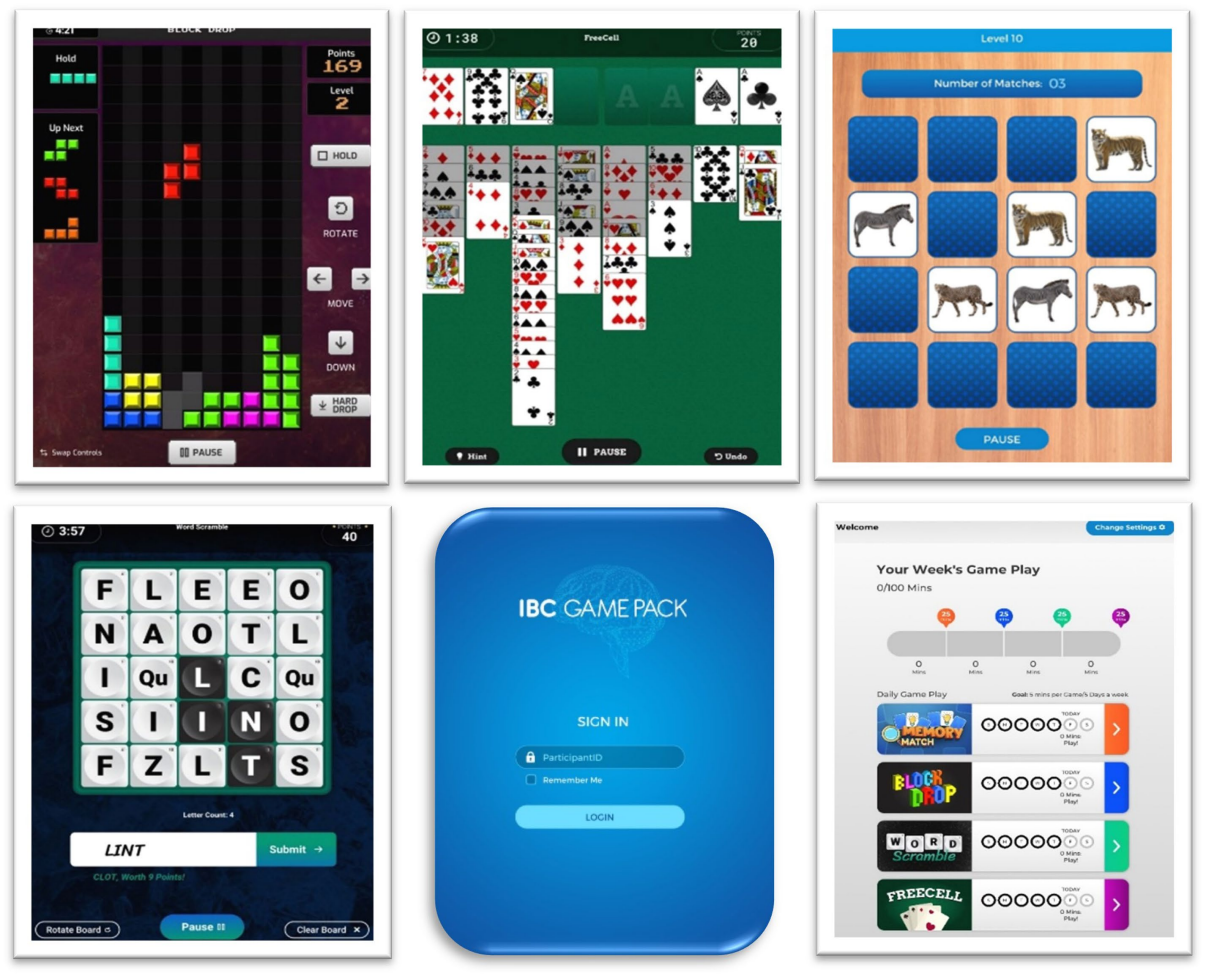
MIND Game Pack platform. (From left to right, top row): ‘Block Drop’, ‘FreeCell’, ‘Memory Match’. (From left to right, bottom row): ‘Word Scramble’, Log-In Screen, Home Screen with compliance visualizations.

The current study investigated the at-home use of MIND GamePack over the course of 4 months. The platform consists of four games: ‘Block Drop’, ‘FreeCell’, ‘Memory Match’, and ‘Word Scramble’ (Figure 1). The MIND GamePack is an iPadOS-based application which passively extracts raw data and custom-defined summary metrics (Supplementary Figure S1) believed to engage key domains of cognition. Data are collected following a game session, either by game completion or early termination (i.e., quitting a game prior to completion). Participants in the study were individually trained to play each of the games via a standardized procedure. At the same visit, all participants were provided with study devices (iPad tablet), pre-loaded with software to take home and were asked to play unsupervised for at least 5 min per game per day for 5 days a week, for a total of 100 weekly minutes. Participants received financial incentive, up to $435 compensation, if they complied with minimum gameplay requirements. This 4-month study had four study intervals: a lead-in period (Day −21 to −14), baseline (Day 1), 6-week follow-up (Day 42), and end-of-study (Day 84).

### Inclusion and exclusion criteria

Eligible participants (Supplementary Table S1) had to meet the following criteria: ages of 55–90, at least a high school diploma or equivalent level of education, native English speaker, Montreal Cognitive Assessment (MoCA) (34) score of 26 or higher, Geriatric Depression Scale (GDS) (35) score of 9 or lower indicating no more than sub-clinical depressive symptomatology, and qualitative evaluation of the Columbia Suicide Severity Rating Scale (C-SSRS) indicating psychiatric stability.

### Games and game features

The MIND GamePack includes four games. Block Drop (e.g., *Tetris*) is a dynamic puzzle game where players control descending blocks of varying 4-square geometric shapes (Supplementary Figure S2), with the goal of aligning blocks in a continuous row and clearing as many rows as possible. To achieve this within 5 min, players must touch a sensitive user-interface (UI) to move and rotate blocks with up, down, right, and left arrow buttons, with the goal of filling gaps on the playing field. When all gaps within a row are filled, the row is cleared. For increased strategy, players may also “hold” descending blocks for later use. For the analysis of Block Drop summary metrics, we extracted the number of lines cleared within a completed 5-min game session. The number of lines cleared is moderately correlated with Trail Making Test A (*r* = 0.5), which is a test commonly measuring motor speed and attention (36).

FreeCell is a derivative of the traditional *Solitaire* card game in which all cards of a 52-card deck are dealt to the player. The goal is to stack all cards in ascending order by suit on the *foundations* pile. To accomplish this, the player may move single (or multiple) cards between the *foundations* pile, four free cells, and the playing field. The player may use their own strategy or request ‘hints’ to solve the “puzzle” in an untimed format. FreeCell is further complicated by employing different rulesets for different piles; in the MIND GamePack version of FreeCell, if the player makes an “incorrect move” the move is withdrawn, the player is notified of the correct ruleset for placement, and they are allowed to try again to advance the puzzle. In analysis of the data generated from FreeCell, we calculated the ratio of correct moves by dividing the number of correct (i.e., non-error producing) moves by the total number of moves per session. The ratio of correct moves is moderately correlated with Trail Making Test B (*r* = 0.5), a test commonly used to measure executive function (36).

Memory Match (like *Concentration*) is a simulated card game where players are given a prespecified amount of time to memorize a matrix of cards before all are turned over. The player must then select match-pairs from memory in an untimed format. Depending on the level selection, the matrix size can range from [2×2] to [8×8], consisting of 2 and 32 matches, respectively. In the case of Memory Match, we calculated a percent accuracy score by dividing the number of correctly selected match-pairs by the total number of flipped cards per session. Percent accuracy score was normalized based on difficulty levels defined by matrix size (i.e., ranging from Level 1–26). Precent accuracy score is moderately correlated with California Verbal Learning Test® Third Edition (CVLT3) Short Delay Free Recall (SDFR) test (*r* = 0.5) and Long Delay Free Recall (LDFR) test (*r* = 0.6). CVLT3 SDFR and LDFR tests are used for assessing short and long-term memory recall (37).

Lastly, Word Scramble (like *Boggle*), is a timed puzzle game in which players are presented with a 5×5 randomized matrix of letters (including the phoneme ‘qu’). By selecting individual letters vertically, horizontally, or diagonally (or a combination thereof), players are tasked with finding as many words as possible (3 letters or longer) within a time limit of 5 min. Players can pause, rotate the board, and clear their current selection of letters as a function of the game. For Word Scramble, we calculated a normalized word-found score by determining the number of possible words on each board (range: 40–7,000) using an augmented dictionary of the ENABLE1 Word List (i.e., Enhanced North American Benchmark LExicon) compiled for public domain. We normalized the word-found score by comparing it to the potential number of words available on each board, using z-score transformation. Normalized word-found score is moderately correlated with WAIS-IV Wechsler Adult Intelligence Scale 4th Edition (WAIS-IV) Subtests Symbol Search Total Score (*r* = 0.6) and Delis-Kaplan Executive Function System (DKEFS) Verbal Fluency Letter Fluency Scaled Score (*r* = 0.4). WAIS-IV symbol search is a test of information processing speed (38) and visual perception while DKEFS verbal fluency letter fluency test is a test of semantic abilities (39).

### Compliance survey

A compliance survey was conducted at week 6 and end-of-study (i.e., week 12) to understand participants’ tolerability and experience with the MIND GamePack (Supplementary Table S2). Interview questions included assessing the burden of game play time requirements, game preference, and general experience of using the platform. In the survey, participants were asked to rank their “favorite” games on a scale of 1–4, with 1 indicating their favorite game and 4 indicating their least favorite game at four study intervals.

### Subjective and environmental factors

Six subjective and environmental factors were identified from the literature that might potentially influence gameplay routines. Only at the first login of each day of play, participants completed a survey within the iPad program before game sessions, rating their sleep quality, energy level, mood, and subjective memory and thinking ability that day on a 1–5 Likert scale. One-item mood assessments have shown sensitivity in detecting changes before and during life events (40). At each login for game play, participants also indicated where they were playing the game (i.e., at home or elsewhere) and whether there were potential distractions in their environment (yes/no) (Supplementary Figure S3).

### Data architecture

**Figure 2:**
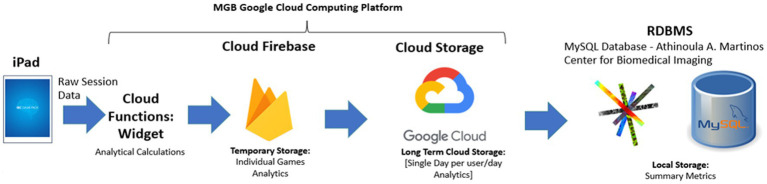
Data architecture of MIND GamePack.

The *Google Cloud Computing Platform* (GCP) was used in the MIND GamePack to achieve wireless data transmission, aggregation, analysis, and back-up. Upon completion of both *individual game sessions* and *daily surveys,* and successful connection to a stable Wi-Fi connection, de-identified participant game data were sent to *Cloud Firestore* per session per user. Transmitted data were analyzed within the GCP, annotated into raw data, and held for daily archiving within the GCP cloud bucket storage. Data were then archived per day per user into JavaScript Object Notation (JSON) files for query in bucket storage. Further, data in this format were then backed up to a local open-source relational database management system (RDBMS) at Massachusetts General Hospital (MGH) (Figure 2).

### Statistical analysis

Gameplay time and number of game sessions were calculated per participant per day over 4 months. A linear generalized estimating equation (GEE) model was utilized to evaluate the changes in gameplay time and number of sessions (41). To estimate how long it may take for game performance to plateau, a regression tree model was used with the outcome being the performance of each game (42). The regression tree model calculates the mean squared error (MSE) for two chunks of time series and identifies the day $\hat{r_{i}}$ when the least MSE was observed (i.e., the day best splitting the time series data). There are three steps with the regression tree model.


$$T\left(r\right)=\underset{x_{i}\leq r}{\sum }{\left(y_{i}-{\hat{m}}_{1}\right)}^{2}+\underset{x_{i}>r}{\sum }{\left(y_{i}-{\hat{m}}_{2}\right)}^{2}$$

- For each participant’s data, sort $\left(x_{i},y_{i}\right)$ by $x_{i}$ in ascending order and set $r_{i}=\frac{1}{2}\left(x_{i}+x_{i+1}\right)$ for i=1,…,n−1.
- The sum of squared distance is minimized by the mean, so for each $r_{i}$, compute
  $${\hat{m}}_{1,i}=\frac{1}{i}\overset{i}{\underset{j=1}{\sum }}y_{j}$$

$${\hat{m}}_{2,i}=\frac{1}{n-i}\overset{n}{\underset{j=i+1}{\sum }}y_{j}$$

$$T\left(r_{i}\right)=\overset{i}{\underset{j=1}{\sum }}{\left(y_{i}-{\hat{m}}_{1,i}\right)}^{2}+\overset{n}{\underset{j=i+1}{\sum }}{\left(y_{i}-{\hat{m}}_{2,i}\right)}^{2}$$

- Find $\hat{r_{i}}$ minimizes $T\left(r_{i}\right)\text{.}$

Regression tree models were conducted on all participants for an identified game feature, and then averaged across the sample, to identify the mean time at which said feature plateaued. The standardized mean squared error (MSE) was calculated for each participant during the monitoring period, and then averaged across participants for each monitoring day.

A multinomial logistic GEE model was used to examine changes in game preference ranks and the likelihood of enjoying a specific game over time. Subjective and environmental factors of gameplay were included in GEE models. The four games’ play time, preference, and total game play time were analyzed separately. All the GEE models were adjusted for age, sex, and years of education.

## Results

### Participant demographics

Twenty-nine participants were screened, and 2 participants were ineligible (MoCA score ≤ 26). Twenty-seven participants were enrolled, and 1 withdrew after stating a lack of financial incentive. In total, 26/27 enrolled participants completed the 12-week study (Table 1). Participants were recruited primarily through the Mass General Brigham Rally platform.

**Table 1:** Participant characteristics (*n* = 26).

Age in years [mean (SD)]	71.87 (8.63)
Female [*n* (%)]	19 (73.08)
Gender identity as female [*n* (%)]	20 (76.92)
Years of education [mean (SD)]	16.59 (1.61)
Race [*n* (%)]	
White-Caucasian/European	24 (92.31)
White-Arabic/North African	1 (3.85)
African American	1 (3.85)
Right-hand dominance [*n* (%)]	23 (88.46)

### Feasibility

**Figure 3:**
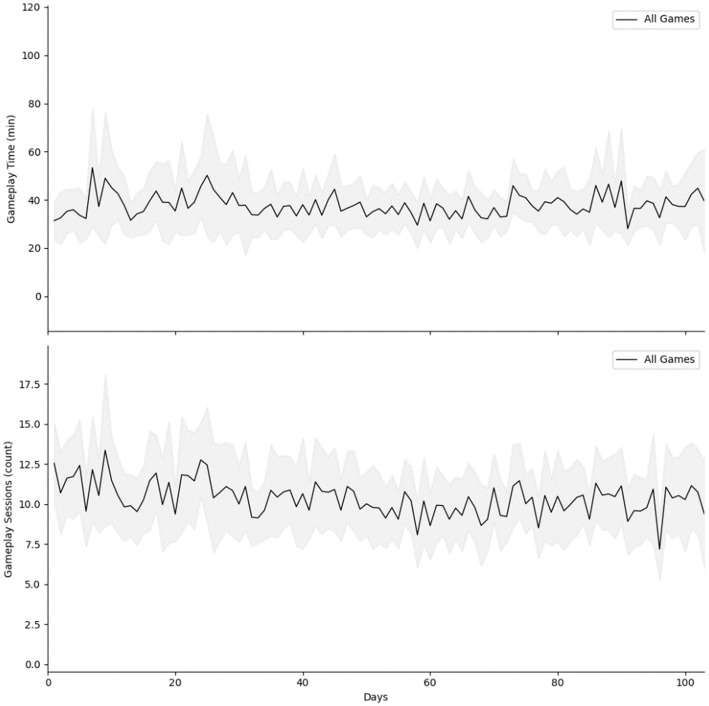
Gameplay time and sessions across days.

A total of 1946 days of data were collected across 26 participants. Over the four-month study period, GEE models revealed total gameplay time (*β* = −0.005, value of *p* = 0.94) and the total played sessions (*β* = −0.01, value of *p* = 0.19) remained stable (Table 2), with an average compliance rate of 91% (SD = 11%). On average, participants played for at least 100 min per week for 11 out of 12 weeks. Participants played on average 3.5 h per week (SD = 2.2) which was approximately 2 h above minimum requested compliance (Figure 3). Table 2 presents the results of GEE models. The analysis revealed a significant association between self-reported memory and thinking ability and total gameplay time and sessions. Specifically, a point increase in daily self-reported memory and thinking ability was associated with 2 min more (or 0.34 session) of game play. There was a significant association between self-reported distractions and the number of game sessions played that day (*p* = 0.01). Participants played 1.3 more sessions if they were in an environment with distractions from people and things in their environment.

**Table 2:** Total gameplay and subjective and environmental factors.

	Duration of play		Number of sessions	
	*β*	*p*	*β*	*p*
Intercept	40.96	0.42	10.70	0.27
Age	0.30	0.52	0.08	0.40
Female	0.82	0.93	1.55	0.43
Education	−1.94	0.42	−0.50	0.43
Days	−0.005	0.94	−0.01	0.19
Daily self-reported survey				
Memory and thinking ability	2.00	0.02*	0.34	0.03*
Mood	0.29	0.76	0.12	0.51
Energy	0.33	0.75	0.01	0.95
Sleep	−0.72	0.44	0.03	0.85
Location (outside the home)	−6.87	0.27	−1.36	0.18
Distractions (from people or things)	6.05	0.17	1.34	0.01*

*Significance *p* < 0.05.

**Figure 4:**
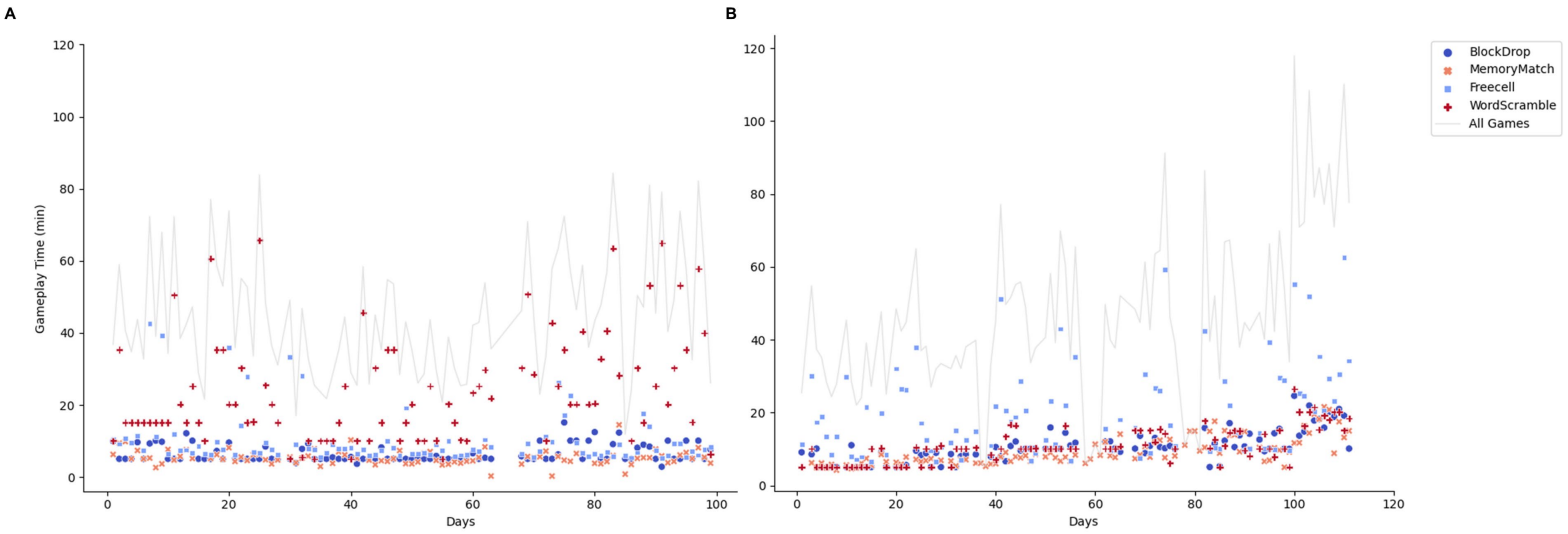
Case examples of gameplay time across days. **(A)** Gameplay time remained stable across 100 days. Word Scramble was played longer than other three games. **(B)** Gameplay time showed a trend of increase. FreeCell was played longer than other three games.

Among the four games, Block Drop, and FreeCell play time and number of played sessions remained stable throughout the study. The number of Memory Match sessions decreased over time (*p* = 0.01), although play time did not significantly decrease (*p* = 0.26) (Table 3). Word Scramble gameplay time (*p* = 0.03) and number of played sessions (*p* = 0.03) showed significant increases over the study period, indicating an approximately increase of 1 min in playtime per week (or 0.2 session). Figure 4 shows the amount of play time for each game from two participants.

**Table 3:** Changes in play time (in minutes) and sessions (in counts) across games.

	Total games				Block Drop				FreeCell				Memory Match				Word Scramble			
	Play time		Sessions		Play time		Sessions		Play time		Sessions		Play time		Sessions		Play time		Sessions	
	*β*	*p*	*β*	*p*	*β*	*p*	*β*	*p*	*β*	*p*	*β*	*p*	*β*	*p*	*β*	*p*	*β*	*p*	*β*	*p*
Intercept	263.51	0.28	71.99	0.22	65.16	0.40	16.59	0.32	151.50	0.26	17.20	0.07	24.34	0.37	29.72	0.37	136.11	0.31	27.45	0.30
Age	2.17	0.29	0.53	0.33	−0.07	0.91	0.04	0.86	1.18	0.45	−0.08	0.50	0.63	0.08	0.42	0.21	0.55	0.39	0.09	0.47
Female	30.62	0.50	14.54	0.17	11.96	0.31	2.45	0.38	−24.81	0.34	0.95	0.57	14.96	<0.01*	5.68	0.46	25.79	0.06	5.28	0.06
Education	−16.96	0.30	−3.49	0.41	−1.32	0.79	−0.62	0.62	−8.08	0.28	−0.21	0.72	−2.65	0.19	−1.84	0.44	−8.82	0.25	−1.71	0.27
Week	0.54	0.86	−0.39	0.38	0.13	0.84	0.02	0.89	0.27	0.90	0.09	0.55	0.13	0.61	−0.50	0.01*	0.88	0.03*	0.21	0.03*

*Significance *p* < 0.05.

### Learning and time to plateau

**Figure 5:**
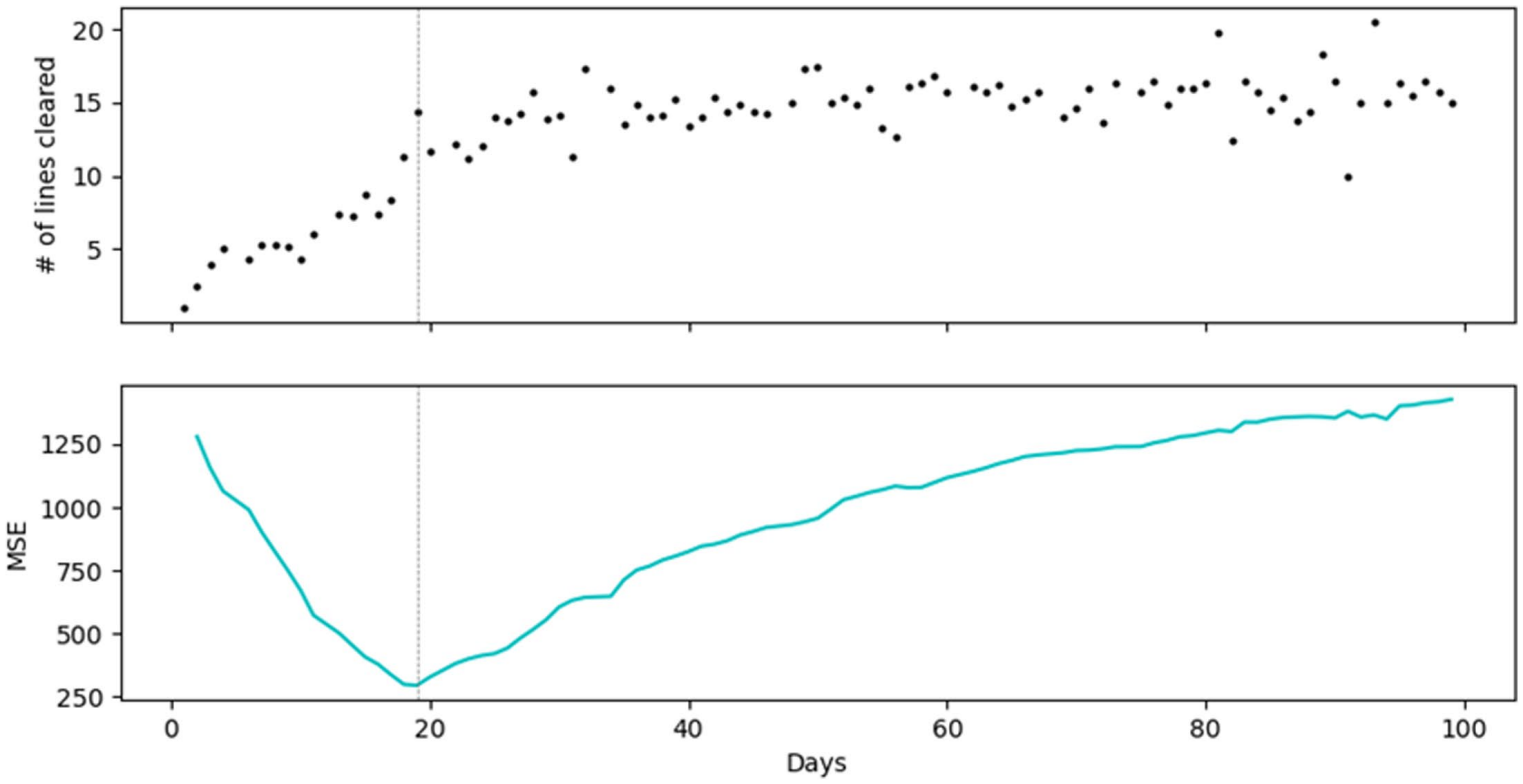
A case example of regression tree model of number of lines cleared in Block Drop. The scatter plot shows the number of lines cleared in Block Drop per day. The line plot shows the MSE for each split in the regression tree models. Result of the regression tree model suggested the participant’ game performance plateaued around day 19.

**Figure 6:**
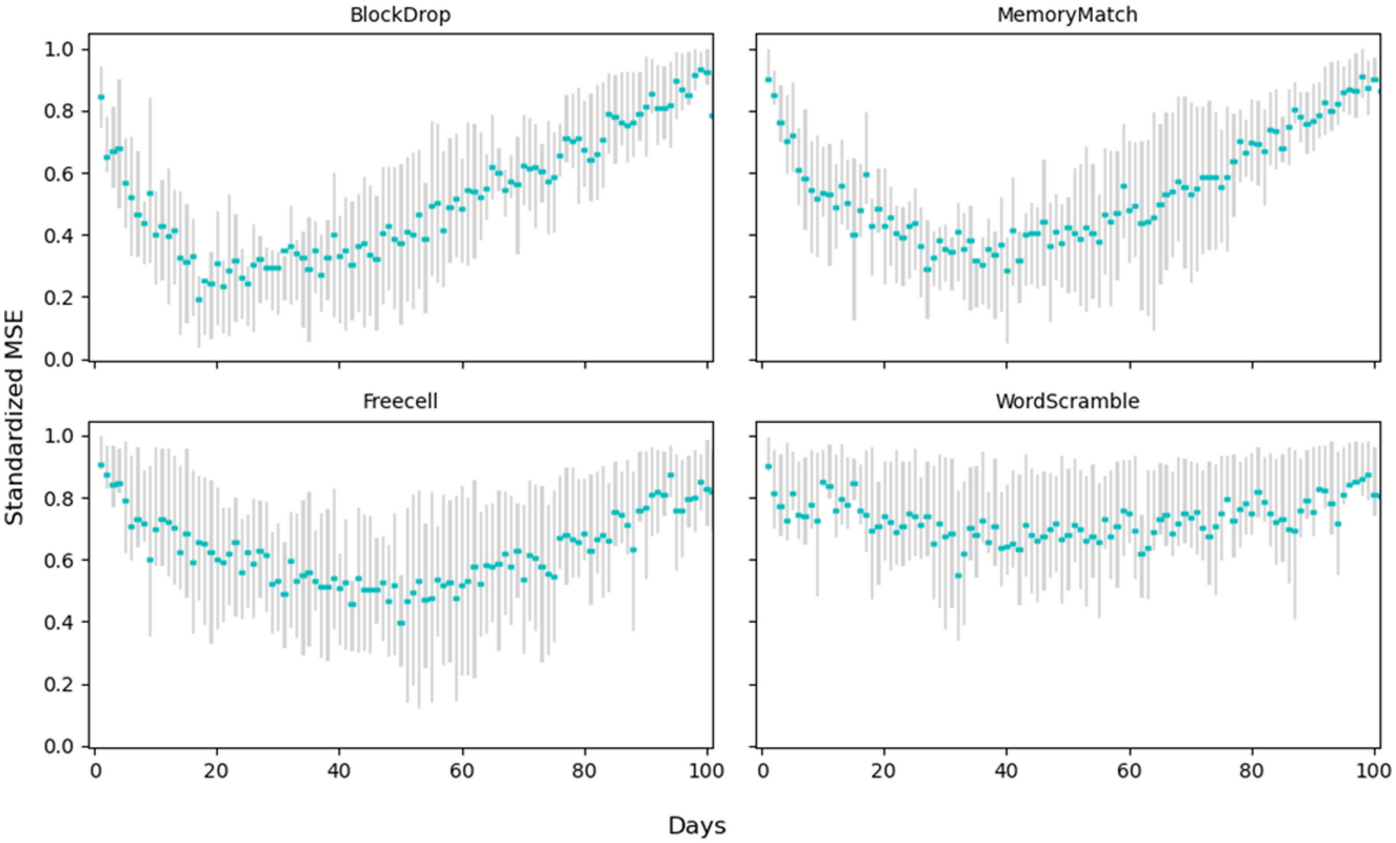
Averaged standardized MSE across 4 games (*n*=26).

The results of the regression tree models are presented in Table 4. Our analysis revealed that for Block Drop, the trajectory of average number of lines cleared plateaued after approximately 3 weeks of gameplay. For FreeCell, the trajectory of the average ratio of the valid moves feature plateaued after 5 weeks of gameplay. For Memory Match, the trajectory of the average normalized percent accuracy score plateaued after approximately 6 weeks of gameplay. For Word Scramble, the trajectory of the average normalized word-found score plateaued after approximately 4 weeks of gameplay. Figure 5 demonstrates the regression tree model and the use of MSE to determine the day when the number of lines cleared plateaued for one case example. Figure 6 presents the averaged standardized MSE for each day of the 4 games throughout the study period.

**Table 4:** Estimated days that a game performance plateaued.

Game	Feature	Description	Average days plateaued (mean ± SD)	Notes
Block Drop	Number of lines cleared	Number of lines cleared in each completed session	21.5 ± 11.8 days	Three participants did not have enough completed sessions (<10 days).
FreeCell	Ratio of valid moves	Number of valid moves/ (Number of valid moves + Number of invalid moves)	34.5 ± 22.4 days	
Memory Match	Normalized percent accuracy	Number of correct cards/ Number of flipped cards, normalized by game difficulties	39.1 ± 16.9 days	
Word Scramble	Normalized word found score	Number of words found/ Number of possible words, normalized by game difficulties in each completed board	26.3 ± 15.8 days	

### Compliance

Table 5 lists selected quotes from participants from the compliance survey in which participants were asked how difficult/easy the MIND GamePack was to incorporate into their daily routine. Participants reported that the current study design was well-tolerated and easy to incorporate in their daily routines. Two participants stated they wanted to continue playing the games after the study concluded. One participant commented that this set of games should be available to everyone participating in research.

**Table 5:** Quotes from the compliance survey.

Aspect	Age	Sex	Quotes
Routines	63	Male	*“Very easy –**I’m routine driven and I just set it up and it happens**….I experiment trying to play early morning, afternoon, and evening – I vary my play.”*
	66	Female	*“Easy – I experimented at the beginning playing two in the morning and two in the afternoon. But now I play them all after breakfast…yes**I do it after my morning coffee and breakfast**.”*
Burden	57	Female	*”**I play a lot of different games and enjoy them**. I have the same ranking of my favorites, but**I sort of incorporate it into my day**. Even Memorial weekend we were gone to Maine. It was easy to change the games and get onto Wi-Fi.”*
General platform feedback	63	Male	*”**The UI is really good quality**. I am a developer- I’m impressed.”*
	74	Female	*“I like the sound effects of the games.**I liked the Block Drop when you get 4 and it goes pheww**…”*
	89	Male	*“It’s interesting, I found the animal ones* [i.e. *levels*] *to be complex and the drinks were hard –**a myriad of*** ***different shaped glasses and colors**.”*
Overall experience	57	Female	*“I thought it was kind of fun. I feel like some of the games**I want to download on my iPad.**”*
	89	Male	*“It was an interesting challenge overall.**Something there for everyone.**”*
	70	Female	*“I really enjoyed,**I’m kind of sad it’s over. I want to play some more.**”*

## Discussion

The current study evaluated the feasibility of the MIND GamePack as a game-based tool for daily assessment of cognitive function in older adults. Both quantitative and qualitative data indicated that participants found the games enjoyable and played them consistently over the 4-month period. Participants had a high compliance rate, averaging at 91%, suggesting that, on average, they were compliant with the study requirements for 11 out of the 12 weeks. Game performance stabilized after 22–39 days (3–6 weeks), reflecting a spectrum of diverse learning processes between participants. Such data can be useful in monitoring subtle, yet personal, changes in cognition in day-to-day lives. The platform includes short daily surveys to capture subjective and environmental factors which may further impact game play. Feasibility data supports the potential of this game-based platform for endpoints in future longitudinal and interventional research in aging and ADRD.

Before using the MIND GamePack in clinical research, it is essential to have characterized the learning curves and plateaus of game performance. If participants keep improving over time, as might occur with “brain exercises,” it becomes difficult to distinguish if improvement is due to intervention or natural learning and improvement processes. We adopted a novel approach using regression tree models to estimate the time when game performance plateaued. Despite variations in the features of different games, the average time-to-plateau for participants was approximately 3–6 weeks. This information can be useful in designing future clinical trials that incorporate the MIND GamePack. To ensure accurate assessment, investigators may consider allowing at least a 1 month of lead-in period before baseline assessment and administering an intervention to participants.

The observed differences in the average time-to-plateau of game features may be attributed to the different cognitive demands of each game. For example, Block Drop requires visuomotor and visuospatial abilities, which may be improved upon more expeditiously compared to other cognitive domains such as memory (Memory Match) or language (Word Scramble). To better understand the concurrent validity of game features, future studies will explore the correlation between the learning rates of these features and conventional neuropsychological tests.

In the current design, participants were asked to play all four games 5 min per day, 5 days a week. Understanding game preferences and levels of enjoyment in playing games is critical to ensuring future compliance and game selection. Because we added the gaming preference survey after recruitment and had a limited sample size, we recognize this is an important area to explore in the future. Validated surveys of participant experience are needed to fully capture user experience and the likelihood of participating in clinical trials with the MIND GamePack.

Our study underscores the importance of assessing both subjective and environmental factors that may affect game play and interpretation of play data. Our findings of better self-reported memory and thinking ability prior to daily gameplay contribute significantly to more gameplay time and higher frequency suggests value for regular assessments to capture cognitive performance on both “good” and “bad” days. We also observed a positive correlation between the number of gameplay sessions and the presence of distractions from other people and things in their environment. This association may be attributed to increased social interactions and discussions about games with family and friends, which may have led to more frequent gameplay sessions. Another explanation might be participants played more to compensate for the time they were distracted.

### Limitations

The MIND GamePack is an easy-to-use, accessible, and cost-effective solution for dense semi-passive monitoring of cognition in aging and ADRD longitudinal research. However, the platform has some limitations that should be considered. Being unsupervised, one possibility is that participants could allow others to play despite their instruction not to permit anyone else to play the games. For the current study, only one participant indicated they let another person, a family member, play a single session. Second, participants may have preferences for certain games and may choose not to play others, which could limit the evidence for learning and make it challenging to identify meaningful time points using a regression tree model. The brief survey of self-reported memory and thinking ability, mood, sleep, and energy has not been formally validated. However, this survey serves as a valuable tool for exploring potential covariates when employing game-based assessments to gauge cognitive variability. Finally, it is important to note that the current study focused on older adults with intact cognition. Ongoing research is exploring the use of the MIND GamePack with older adults with mild cognitive impairment (MCI) or mild dementia due to AD.

### Future directions

Additional research is exploring the use of MIND GamePack in older adults with MCI and mild-stage dementia due to AD. This will allow us to assess whether individuals with memory problems are able to play the games and adhere to the protocol. The construct validity of game features can be better described using a more heterogeneous population across the ADRD continuum. The cognitive domains for validating the MIND GamePack could include global cognition, memory, motor, visuospatial, language, and executive function. Additionally, we suggest developing more features to assess learning and comparing the learning performance of game features across different levels of cognitive impairment to gain a better understanding of the natural trajectories of learning with and without ADRD. The availability of multiple features will support the application of machine learning and linguistic techniques to characterize cognitive variability and heterogeneity. Such insights could be used in future ADRD research to enhance the use of the MIND GamePack as a robust complementary assessment tool for evaluating disease progression and the effectiveness of interventions being evaluated for stabilization or even symptomatic improvement.

## Data availability statement

Data is available from the corresponding author on reasonable request. Requests to access the datasets should be directed to SEA, SEARNOLD@mgh.harvard.edu.

## Ethics statement

The studies involving humans were approved by the institutional review board (IRB) (MGB #2021P000144). The studies were conducted in accordance with the local legislation and institutional requirements. The participants provided their written informed consent to participate in this study.

## Author contributions

NS: Conceptualization, Data curation, Funding acquisition, Investigation, Methodology, Project administration, Visualization, Writing – review & editing. C-YW: Formal analysis, Methodology, Visualization, Writing – original draft, Writing – review & editing. JaG: Data curation, Project administration, Validation, Visualization, Writing – review & editing. TD: Formal analysis, Validation, Visualization, Writing – review & editing. AF: Data curation, Project administration, Writing – review & editing. JeG: Conceptualization, Funding acquisition, Project administration, Writing – review & editing. RK: Formal analysis, Software, Validation, Visualization, Writing – review & editing. BR: Data curation, Formal analysis, Methodology, Software, Writing – review & editing. MR: Data curation, Project administration, Supervision, Writing – review & editing. LS: Data curation, Project administration, Writing – review & editing. XW: Writing – review & editing. CY: Project administration, Writing – review & editing. LY: Writing – review & editing. HHD: Supervision, Writing – review & editing. SEA: Conceptualization, Funding acquisition, Investigation, Methodology, Supervision, Writing – review & editing.

## References

[1] Jacobs DM Sano M Dooneief G Marder K Bell KL Stern Y. Neuropsychological detection and characterization of preclinical Alzheimer’s disease. Neurology. (1995) 45:957–62. doi: 10.1212/WNL.45.5.957, PMID: 7746414

[2] Weintraub S Besser L Dodge HH Teylan M Ferris S Goldstein FC. Version 3 of the Alzheimer disease Centers’ neuropsychological test battery in the uniform data set (UDS). Alzheimer Dis Assoc Disord. (2018) 32:10–7. doi: 10.1097/WAD.0000000000000223, PMID: 29240561 PMC5821520

[3] Visser P Scheltens P Verhey F. Do MCI criteria in drug trials accurately identify subjects with predementia Alzheimer’s disease? J Neurol Neurosurg Psychiatry. (2005) 76:1348–54. doi: 10.1136/jnnp.2004.047720, PMID: 16170074 PMC1739362

[4] Wilson RS Segawa E Boyle PA Anagnos SE Hizel LP Bennett DA. The natural history of cognitive decline in Alzheimer’s disease. Psychol Aging. (2012) 27:1008. doi: 10.1037/a0029857, PMID: 22946521 PMC3534850

[5] Scannell JW Blanckley A Boldon H Warrington B. Diagnosing the decline in pharmaceutical R&D efficiency. Nat Rev Drug Discov. (2012) 11:191–200. doi: 10.1038/nrd3681, PMID: 22378269

[6] Scott TJ O’Connor AC Link AN Beaulieu TJ. Economic analysis of opportunities to accelerate Alzheimer’s disease research and development. Ann N Y Acad Sci. (2014) 1313:17–34. doi: 10.1111/nyas.12417, PMID: 24673372 PMC4285871

[7] Cummings JL Goldman DP Simmons-Stern NR Ponton E. The costs of developing treatments for Alzheimer’s disease: a retrospective exploration. Alzheimers Dement. (2022) 18:469–77. doi: 10.1002/alz.12450 34581499 PMC8940715

[8] Anderson RM Hadjichrysanthou C Evans S Wong MM. Why do so many clinical trials of therapies for Alzheimer’s disease fail? Lancet Lond Engl. (2017) 390:2327–9. doi: 10.1016/S0140-6736(17)32399-1 29185425

[9] van Dyck CH Swanson CJ Aisen P Bateman RJ Chen C Gee M. Lecanemab in early Alzheimer’s disease. N Engl J Med. (2023) 388:9–21. doi: 10.1056/NEJMoa2212948, PMID: 36449413

[10] Beattie Z Miller LM Almirola C Au-Yeung W-TM Bernard H Cosgrove KE. The collaborative aging research using technology initiative: an open, sharable, technology-agnostic platform for the research community. Digit Biomark. (2020) 4:100–18. doi: 10.1159/000512208 33442584 PMC7768162

[11] Kaye J Reynolds C Bowman M Sharma N Riley T Golonka O. Methodology for establishing a community-wide life laboratory for capturing unobtrusive and continuous remote activity and health data. J Vis Exp. (2018) 2018:1–10. doi: 10.3791/56942 PMC6126551 30102277

[12] U.S. Department of Health and Human Services Food and Drug Administration. Enrichment strategies for clinical trials to support approval of human drugs and biological products guidance for industry. Silver Spring, MD (2019).

[13] Dodge HH Zhu J Mattek NC Austin D Kornfeld J Kaye JA. Use of high-frequency in-home monitoring data may reduce sample sizes needed in clinical trials. PLoS One. (2015) 10:e0138095. doi: 10.1371/journal.pone.0138095, PMID: 26379170 PMC4574479

[14] Wu C-Y Beattie Z Mattek N Sharma N Kaye J Dodge HH. Reproducibility and replicability of high-frequency, in-home digital biomarkers in reducing sample sizes for clinical trials. Alzheimers Dement Transl Res Clin Interv. (2021) 7:e12220. doi: 10.1002/trc2.12220 PMC8719347 35005204

[15] Wu C-Y Dodge HH Reynolds C Barnes LL Silbert LC Lim MM. In-home mobility frequency and stability in older adults living alone with or without MCI: introduction of new metrics. Front Digit Health. (2021) 3:764510. doi: 10.3389/fdgth.2021.764510, PMID: 34766104 PMC8575720

[16] Wang S James Hung HM O’Neill RT. Adaptive patient enrichment designs in therapeutic trials. Biom J J Math Methods Biosci. (2009) 51:358–74. doi: 10.1002/bimj.200900003 19358222

[17] Dorociak KE Mattek N Lee J Leese MI Bouranis N Imtiaz D. The survey for memory, attention, and reaction time (SMART): development and validation of a brief web-based measure of cognition for older adults. Gerontology. (2021) 67:740–52. doi: 10.1159/000514871, PMID: 33827088 PMC8494835

[18] Au-Yeung W-TM Kaye JA Beattie Z Step count standardization: validation of step counts from the withings activite using PiezoRxD and wGT3X-BT. 42nd annual international conference of the IEEE engineering in Medicine & Biology Society (EMBC). IEEE. (2020) 2020:4608–11. doi: 10.1109/EMBC44109.2020.9176511, PMID: 33019020 PMC7759156

[19] Calamia M Markon K Tranel D. Scoring higher the second time around: meta-analyses of practice effects in neuropsychological assessment. Clin Neuropsychol. (2012) 26:543–70. doi: 10.1080/13854046.2012.680913, PMID: 22540222

[20] Goldberg TE Harvey PD Wesnes KA Snyder PJ Schneider LS. Practice effects due to serial cognitive assessment: implications for preclinical Alzheimer’s disease randomized controlled trials. Alzheimers Dement Amst Neth. (2015) 1:103–11. doi: 10.1016/j.dadm.2014.11.003 PMC4876902 27239497

[21] Ammal SM Jayashree LS. Chapter 10 - early detection of cognitive impairment of elders using wearable sensors In: Peter JD Fernandes SL, editors. Systems simulation and Modeling for cloud computing and big data applications. Advances in ubiquitous sensing applications for healthcare: Academic Press (2020). 147–59.

[22] Godfrey A Brodie M van Schooten KS Nouredanesh M Stuart S Robinson L. Inertial wearables as pragmatic tools in dementia. Maturitas. (2019) 127:12–7. doi: 10.1016/j.maturitas.2019.05.010, PMID: 31351515

[23] Low CA. Harnessing consumer smartphone and wearable sensors for clinical cancer research. NPJ Digit Med. (2020) 3:1–7. doi: 10.1038/s41746-020-00351-x 33134557 PMC7591557

[24] Gielis K Abeele M-EV De Croon R Dierick P Ferreira-Brito F Van Assche L. Dissecting digital card games to yield digital biomarkers for the assessment of mild cognitive impairment: methodological approach and exploratory study. JMIR Serious Games. (2021) 9:e18359. doi: 10.2196/18359, PMID: 34734825 PMC8603181

[25] Gielis K Brito F Tournoy J Vanden AV. Can Card Games Be Used to Assess Mild Cognitive Impairment? A Study of Klondike Solitaire and Cognitive Functions. In Extended Abstracts Publication of the Annual Symposium on Computer-Human Interaction in Play (CHI PLAY ‘17 Extended Abstracts). New York, NY, USA: Association for Computing Machinery. (2017). 269–76.

[26] Jimison H Pavel M McKanna J Pavel J. Unobtrusive monitoring of computer interactions to detect cognitive status in elders. IEEE Trans Inf Technol Biomed. (2004) 8:248–52. doi: 10.1109/TITB.2004.835539, PMID: 15484429

[27] Landers RN Armstrong MB Collmus AB Mujcic S Blaik J. Theory-driven game-based assessment of general cognitive ability: design theory, measurement, prediction of performance, and test fairness. J Appl Psychol. (2022) 107:1655. doi: 10.1037/apl0000954, PMID: 34672652

[28] De Schutter B. Never too old to play: the appeal of digital games to an older audience. Games Cult. (2011) 6:155–70. doi: 10.1177/1555412010364978

[29] Leduc-McNiven K White B Zheng H McLeod RD Friesen MR. Serious games to assess mild cognitive impairment: ‘the game is the assessment. Res Rev Insights. (2018) 2:1–11. doi: 10.15761/RRI.1000128, PMID: 37276027

[30] Bonnechère B Van Vooren M Bier J-C De Breucker S Van Hove O Van Sint JS. The use of mobile games to assess cognitive function of elderly with and without cognitive impairment. J Alzheimers Dis. (2018) 64:1285–93. doi: 10.3233/JAD-180224, PMID: 29991133

[31] Nef T Chesham A Schütz N Botros AA Vanbellingen T Burgunder J-M. Development and evaluation of maze-like puzzle games to assess cognitive and motor function in aging and neurodegenerative diseases. Front Aging Neurosci. (2020) 12:87. doi: 10.3389/fnagi.2020.00087 32372942 PMC7188385

[32] Tong T Chignell M Lam P Tierney MC Lee J. Designing serious games for cognitive assessment of the elderly. Proceedings of the international symposium on human factors and ergonomics In: Health care. Los Angeles, CA: Sage Publications (2014). 28–35.

[33] McLaughlin PM Sunderland KM Beaton D Binns MA Kwan D Levine B. The quality assurance and quality control protocol for neuropsychological data collection and curation in the Ontario neurodegenerative disease research initiative (ONDRI) study. Assessment. (2021) 28:1267–86. doi: 10.1177/1073191120913933, PMID: 32321297

[34] Nasreddine ZS Phillips NA Bédirian V Charbonneau S Whitehead V Collin I. The Montreal cognitive assessment, MoCA: a brief screening tool for mild cognitive impairment. J Am Geriatr Soc. (2005) 53:695–9. doi: 10.1111/j.1532-5415.2005.53221.x 15817019

[35] Sheikh JI Yesavage JA. Geriatric depression scale (GDS): recent evidence and development of a shorter version. Clin Gerontol J Aging Ment Health. (1986) 5:165–73. doi: 10.1300/J018v05n01_09

[36] Reitan RM. Validity of the trail making test as an indicator of organic brain damage. Percept Mot Skills. (1958) 8:271–6. doi: 10.2466/pms.1958.8.3.271

[37] Delis DC Kramer JH Kaplan E Ober BA. California verbal learning test, third edition (CVLT-3): manual. San Antonio: Pearson (2017).

[38] Wechsler D. WAIS-IV Adminstration and scoring manual. San Antonio: Psychological Corporation (1997).

[39] Delis DC Kaplan E Kramer JH. Delis-Kaplan executive function system. Bloomington: Pearson (2001).

[40] Leese MI Bernstein JPK Dorociak KE Mattek N Wu C-Y Beattie Z. Older adults’ daily activity and mood changes detected during the COVID-19 pandemic using remote unobtrusive monitoring technologies. Innov Aging. (2021) 5:1–9. doi: 10.1093/geroni/igab032 34671706 PMC8499772

[41] Hardin JW Hilbe JM. Generalized estimating equations. New York: CRC press (2012).

[42] Lewis RJ. An introduction to classification and regression tree (CART) analysis. Annual meeting of the society for academic emergency medicine in. San Francisco, California: Citeseer (2000).

