# Supplementary material for: Feasibility of common, enjoyable game play for assessing daily cognitive functioning in older adults
Source: Front Neurol. 2023 Oct 12;14:1258216. doi: 10.3389/fneur.2023.1258216 (PMC10602782; doi:10.3389/fneur.2023.1258216)
Supplement: Supplementary file 1 [file Data_Sheet_1.docx]

Supplementary Material

Feasibility of Common, Enjoyable Game Play for Assessing Daily Cognitive Functioning in Older Adults

Schwab, N.A.^1†^, Wu, C-Y.^1†^, Galler, J.A.^1^, DeRamus, T.^1^, Ford, A.^1^, Gerber, J.^1^, Kitchen, R.^1^, Rashid, B.^1^, Riley, M.M.^1^, Sather, L.^1^, Wang, X.^2^, Young, C.K.^1^, Yang, L.^2^, Dodge, H.H.^1^, Arnold, S.E.^1^*

*** Correspondence:** S.E. Arnold; searnold@mgh.haravard.edu

# Supplementary Figures and Tables

## Supplementary Figures

**Supplementary Figure 1.** Data aggregation


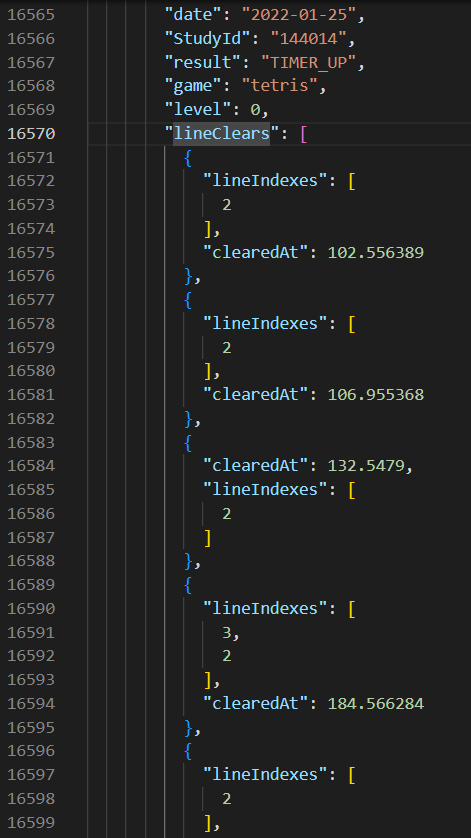


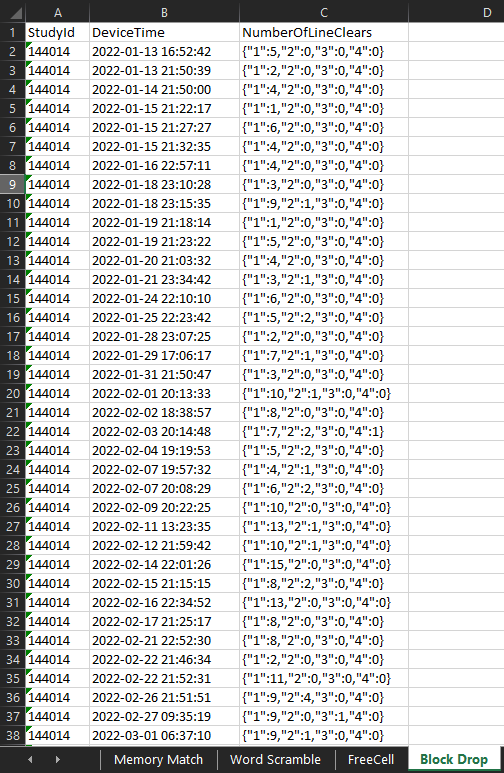


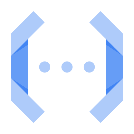


**B.**


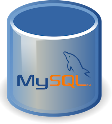

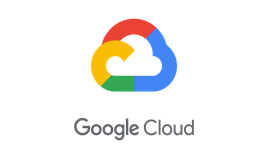


**C.**

**A.**

**(A).** Raw data can be queried by the research team in *GCP Bucket Storage* per user, per day. Raw data pictured (left) is for ‘Block Drop’ lines cleared feature, for a single participant, 144-014. **(B).** *GCP Cloud Functions* sync data to a local RDBMS daily, where researchers can create custom SQL queries to export data. **(C).** The .csv export (right) displays “lines cleared” feature data for participant 144-014 across *many time points* throughout the study*.*

**Supplementary Figure 2.** ‘Block Drop’ shapes

**
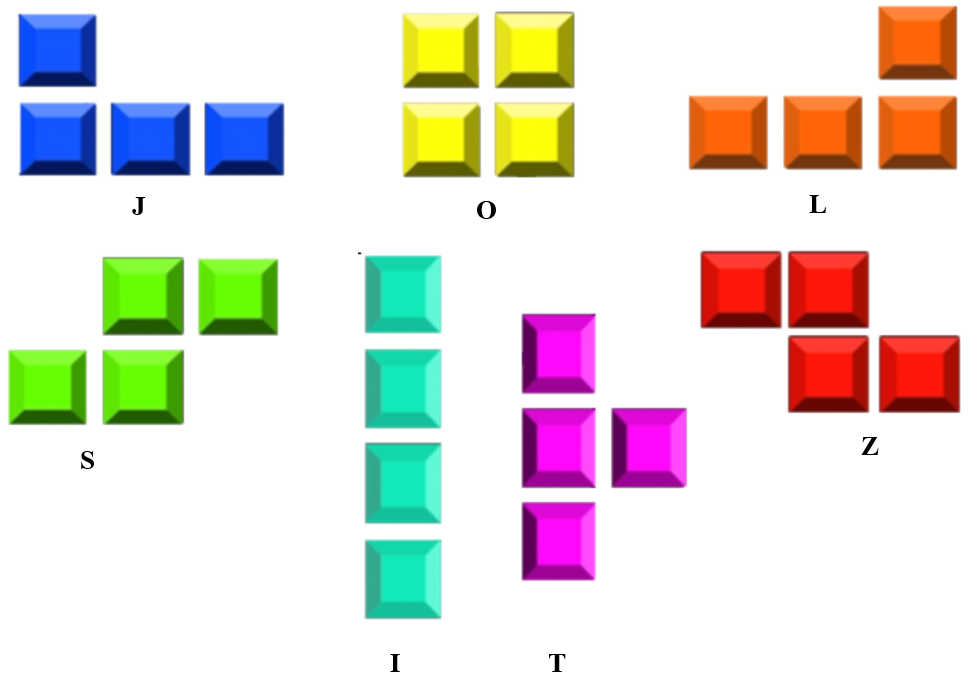
**

**Supplementary Figure 3.** Daily survey questions


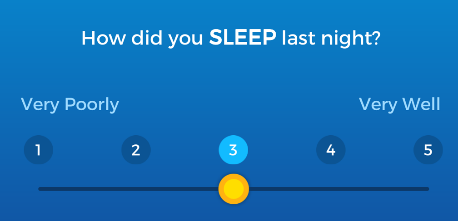

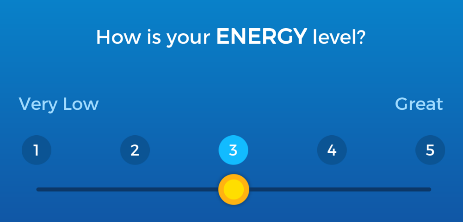

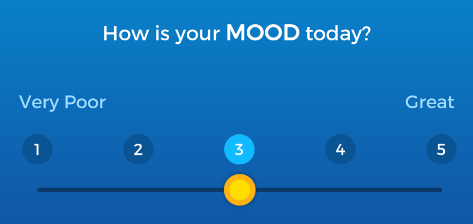

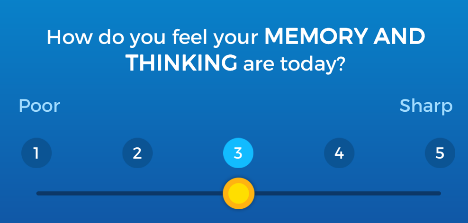

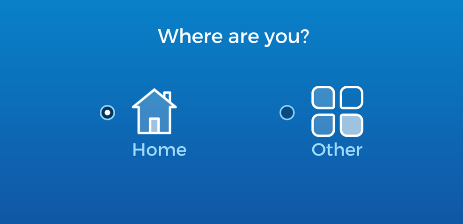

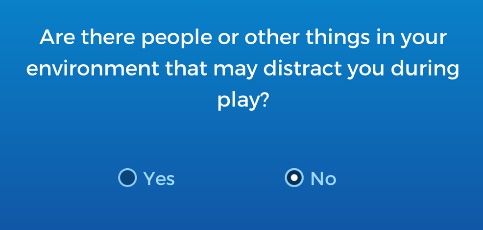


## Supplementary Tables

**Supplementary Table 1.** Inclusion and exclusion criteria

| Inclusion criteria | 1. Ages 55 to 90 inclusive; 2. MoCA score ≥26; 3. Have normal cognitive functioning as defined by a TICS score ≥ 31 and have no subjective cognitive complaints 4. Education level (high school diploma or equivalent), native English speaker, and literacy that indicates participant will be able to complete all assessments according to protocol 5. Ability to provide informed consent 6. Willing and able to complete all assessments and study procedures 7. Geriatric Depression Scale <7 |
| --- | --- |
| Exclusion criteria | 1. Any self-reported specific CNS disease history such as major clinical stroke, brain tumor, normal pressure hydrocephalus, multiple sclerosis, significant head trauma with persistent neurological of cognitive deficits or complaints 2. Any self-reported clinically significant unstable medical condition that could affect safety or compliance with the study and would, in the opinion of the primary investigator, pose a risk to the participant if they were to participate in the study 3. Self-reported major active or chronic unstable psychiatric illness (e.g. depression, bipolar disorder, obsessive compulsive disorder, schizophrenia) within the previous year 4. Current suicidal ideation *or* history of suicide attempt within the last five years 5. History of alcohol or other substance abuse or dependence within the past two years 6. Self-reported laboratory abnormalities in B12, TSH, or other common laboratory parameters that might contribute to cognitive dysfunction or other abnormalities in hematological, hepatic, or renal function tests 7. Current self-reported use of medications with psychoactive properties that may be deleteriously affecting cognition (e.g., anticholinergics, antihistamines, antipsychotics, sedative hypnotics, anxiolytics) 8. Use of other investigational agents or interventions, or participation in clinical studies three months prior to entry and for the duration of the study, unless approved by the investigator 9. History of neurodevelopmental disease (e.g. dyslexia, autism spectrum disorder (ASD), attention deficit hyperactivity disorder (ADHD), etc.), unless approved by the investigator |

**Supplementary Table 2.** Compliance survey

| 1. | How difficult/easy was this to incorporate into your routine so far? |
| --- | --- |
| 2. | Has it been burdensome? |
| 3. | Have you experienced technological issues? |
| 4. | Has anyone else played (e.g. spouse, friend, grandchild)?  If the answer to this question is yes: (1) try to have subject estimate when this occurred and (2) remind subject they are the only person to be playing these games |
| 5. | Which games are you enjoying most/least? |
| 6. | What did you think about the platform overall? Any feedback for improvement? |
| 7. | Any other general feedback? |
